# Comparative study of a liposome and emulsion system with cinnamon essential oil on the quality and proteolysis of refrigerated minced pork

**DOI:** 10.3389/fnut.2024.1341827

**Published:** 2024-05-03

**Authors:** Kegang Wu, Tong Zhang, Xianghua Chai, Pingping Wang, Xuejuan Duan

**Affiliations:** School of Chemical Engineering and Light Industry, Guangdong University of Technology, Guangzhou, China

**Keywords:** liposome, emulsion, cinnamon essential oil, refrigerated minced pork, proteolytic changes

## Abstract

Essential oils have been recognized for their strong antibacterial property, making them an innovative approach for preserving meat. However, their chemical instability and direct impact on meat proteins limit their application. To overcome these limitations, various loading systems have been explored. This study aimed to compare the effect of cinnamon essential oil (CEO) loaded in a liposome and emulsion system on the proteolysis of minced pork and to evaluate the advantages of each delivery system in preventing microorganism-induced quality deterioration of meat. Minced pork treated with CEO-liposomes exhibited lower pH, total volatile basic nitrogen (TVB-N), and total viable count (TVC) values than CEO-emulsions and provided better protection against microorganisms. SDS-polyacrylamide gel electrophoresis (PAGE) analysis confirmed that CEO-liposome was more effective in protecting proteins from degradation. Moreover, CEO-liposome produced lower amount of bitter amino acids and harmful biogenic amines. Antibacterial mechanisms indicated that CEO-liposome exhibited a stronger inhibitory effect against major spoilage bacteria in meat products by increasing cell membrane permeability. The membrane damage was further supported by an increase in conductivity and the leakage of nucleic acids. Compared to the CEO-emulsion system, CEO-liposome emerged as an effective preservative for minced pork. These results provided important theoretical support for using a bioactive compound delivery system to prevent microorganism-induced quality deterioration in meat.

## 1 Introduction

Minced pork is a popular choice in the food industry due to its fresh taste and versatility. It can be shaped into patties, burgers, meat stuffing for buns, pies, dumplings, and more (1). Therefore, it is a convenient and delicious dietary form for humans. However, minced pork is a highly perishable food due to its larger surface area, which increases the exposure to external elements. Additionally, its high lipid, protein, and moisture content contributes to microbial growth and oxidation (2); therefore, various chem-synthetic antibacterial agents and antioxidants are commonly used to extend the shelf life of fresh meat. However, with the growing demand for natural, healthy, and safe preservatives, naturally antibacterial and antioxidant substances such as basil essential oil, thyme essential oil, and cinnamon essential oil (CEO) have been employed for the preservation of pork (3).

CEO is the main component of cinnamon, which has a wide range of biological properties, including antibacterial, antifungal, antioxidant, and immunoregulation activities (4). However, pure essential oils (EOs) are known to be unstable and sensitive to factors such as light, oxygen, and high temperature. These oils also tend to have low water solubility and can easily impart an unpleasant flavor when used in food (5). Moreover, EOs have to be present in high concentrations to obtain positive antibacterial and antioxidant effects in meat preservation due to the interaction between EOs and the fat-soluble part of the food matrix. Thus, the high concentration of EOs would cause an unpleasant smell and flavor and cause protein degradation in food applications (6). To overcome this limitation and increase the water solubility, stability, and bio-availability of bioactive compounds, a series of delivery systems concentrated on emulsions, nanoparticles, microcapsules, and liposomes have been developed in recent years (7).

Liposomes are prepared by phospholipids for encapsulating bioactive compounds. It can deliver the drug into the cell due to its function of cell membrane fusion but with a low encapsulation rate. Cui et al. (8) reported that the antibacterial ability of clove oil increased after encapsulation by phospholipids. More importantly, the clove oil liposome can protect tofu by forming a protective film around the bacteria. An emulsion can combine hydrophilic substances with hydrophobic substances using a surfactant, thereby preparing a stable loading system. It is regarded as an effective, low-cost, convenient delivery system to increase the dispersivity, stability, and bio-availability of bioactive compounds (9). Snoussi et al. (10) showed that beef treated with Tunisian thyme essential oil nanoemulsion can reduce and inhibit the growth of spoilage microorganisms, thereby extending the shelf-life of meat products. Both the liposome and emulsion loading systems were widely proven to increase the antibacterial and antioxidant abilities of bioactive compounds, which are also beneficial for long-time food preservation (10–12).

However, studies mainly focused on the morphology, distribution, and antibacterial and antioxidant abilities of a single loading system, whereas there are few references for the comparative food preservation effect of the emulsion and liposome with the same amount of bioactive compounds. Moreover, there are no comprehensive conclusions for the strategy of each delivery system to reduce the negative sensory effect by improving core-material utilization in meat preservation. Therefore, this study aims to compare the effects of CEO-liposome and CEO-emulsion on quality changes in minced pork in terms of pH, total volatile basic nitrogen (TVB-N), total viable count (TVC), protein degradation, and a combination of free amino acids (FAAs) and biogenic amines (BAs). This evaluation aims to determine the effects of the liposome and emulsion system loading CEO for the whole preservation process of minced pork, potentially providing important theoretical support for the application of a bioactive compound delivery system in meat preservation.

## 2 Materials and methods

### 2.1 Materials and chemicals

*Escherichia coli* (*E. coli*, ATCC8739) and *Staphylococcus aureus* (*S. aureus*, ATCC6538) were purchased from the Guangdong Institute of Microbiology (Guangzhou, China). Minced pork without fat and connective tissues was obtained from the Nanting market (Guangzhou, China). The SDS- polyacrylamide gel electrophoresis (PAGE) gel preparation kit, marker 130, and marker 245 were purchased from Beijing Solarbio Science and Technology Co., Ltd (Beijing, China). All chemicals purchased are of analytical grade.

### 2.2 The preparation of the CEO-liposome

The CEO-liposome was prepared using the ethanol injection method described by Carine et al. (13) with some modifications. First, phospholipids (10 mg/ml), cholesterol (5 mg/ml), and CEO (5 mg/ml) were dissolved in absolute ethanol by stirring. The obtained mixture was then injected into 20 ml ultrapure water at 54°C. The CEO-liposome would be formed spontaneously after stirring at 300 RPM for 15 min. Finally, the prepared liposome was evaporated to remove ethanol and part of the water by using a rotary evaporator (RE-52 A, Yarong Co. Ltd, Shanghai, China) at 40°C under reduced pressure.

### 2.3 The preparation of CEO-emulsion

The CEO-emulsion was prepared as previously described (14). The chitosan solution (1%, w/v) was prepared with 0.5% acetic acid, and the pectin solution (1%, w/v) was dissolved in deionized water. Both the solutions were stirred at 25°C for 2 h. Subsequently, the chitosan solution and pectin solution were mixed at a ratio of 1:1 as the emulsion matrix. Then, 1% CEO solution (w/w) containing 0.25% Tween 80 was added to the matrix solution and stirred until uniform dispersion. The mixture was homogenized by using a high-speed homogenizer (Guo Hua Electric Instrument Co., Ltd., Changzhou, China) at 10,000 RPM for 2 min to obtain the macroemulsion. Furthermore, the macroemulsion was further emulsified using ultrasound for 7.5 min with 600 W. The drug load of the CEO-liposome and CEO-emulsion is shown in Supplementary Table S1.

### 2.4 The particle size of CEO-liposome and CEO-emulsion

The mean particle diameter was measured using a Nano Zetasizer (BI-200SM, USA) at 25°C. Samples were dispersed in ultrapure water to maintain the scattering light intensity below 300 kcps.

### 2.5 The preparation of conditioned minced pork

Minced pork samples were treated with CEO (0%, 0.05%, 0.10%, 0.15%, and 0.20%) and cooked in water (200 ml) at 100°C for 6 min. All the samples were graded in terms of their taste and smell by 10 member panels. The sensory assessments of samples were evaluated by a subjective hedonic scale from 0 (extremely dislike, not acceptable) to 10 points (like extremely).

After sterilization by ultraviolet irradiation, minced pork was mixed with *E. coli* (10^5^ CFU/ml) and *S. aureus* (10^5^ CFU/ml). Afterward, the minced pork was mixed thoroughly with 0.05% CEO (w/w) (or CEO-liposome and CEO-emulsion equivalents to 0.05% CEO) and dried at 25°C for 5 min. The control group was fresh minced pork without CEO treatment. Then, each closed food-grade plastic box carried the tested minced pork (20 ± 0.2 g) placed in a refrigerator at 4°C in the dark. Three parallel minced pork samples of each treatment group in different closed food-grade plastic boxes were collected and analyzed at 0, 2, 4, 6, 8, and 10 days of storage. Each parallel group was collected from a separate box every 2 days to avoid contamination from repeated sampling.

### 2.6 Physicochemical analysis

#### 2.6.1 pH

Minced pork samples (3 g) were homogenized with 15 ml of 0.1 M NaCl solution () at 9,000 RPM for 2 min, and the residue was then filtered using a filter paper. The pH of the filtrate was determined by using a digital pH meter (PHS-3C; Instruments and Electronics Shanghai Associates Co., Ltd, Shanghai, China) at 25°C (15).

#### 2.6.2 Total volatile basic nitrogen

TVB-N was determined using the method described by GB 5009.228-2016. The TVB-N value was calculated using Equation (1):


(1) $$TVB-N=\frac{(V_{1}-V_{2})\times c\times 14}{m\times \left(\frac{V}{V_{0}}\right)}\times 100$$

where V_1_ is the consumption volume of the hydrochloric acid standard titration solution, V_2_ is the consumption volume in the control group, c is the concentration of the hydrochloric acid standard titration solution, m is the weight of the tested minced pork, V = 10, and V_0_ = 30.

#### 2.6.3 Total viable count

The total viable count (TVC) of minced pork was measured according to the method described in GB 4789.2-2022. Briefly, the minced pork (2 g) was homogenized with 18 ml of the NaCl solution (8.5 g/L), and then the homogenous minced pork (1 ml) with proper 10-fold serial dilutions was mixed with 19 ml plate count agar and poured into plates. The plates were incubated at 37°C for 48 h, and the TVC of bacteria was determined after incubation.

### 2.7 The determination of proteolytic changes in minced pork

#### 2.7.1 SDS-PAGE

Polyacrylamide gel electrophoresis (PAGE) was used to evaluate the changes in minced pork protein contents during 10-day storage. The minced pork (2 g) on 0, 2, 6, and 10 days was homogenized with 18 ml of the protein dissolution liquid (5% SDS, 0.1% 2-hydroxy-1-ethanethiol) at 9,000 RPM for 2 min and incubated in a water bath at 90°C for 1 h, followed by centrifugation at 3,000 *g* for 15 min. The supernatant (10 μl) was mixed with SDS-PAGE loading buffer at a ratio of 1:1.

Stacking gel (5%) and separating gel (12% and 8%) were prepared using an SDS-PAGE gel preparation kit. The spot sampling volume was 10 μl, and ColorMixed Protein Marker (11–245 kd and 15–130 kd for 8% and 12% separating gel, respectively) was used as the molecular weight standard control. A staining solution was used to dye the separation gel for 1 h after electrophoresis and decolor the separation gel using a decoloring solution for 24 h with gentle shaking. The coomassie brilliant blue (2.5 g/L) staining solution comprised water/methanol/acetic acid in a volumetric ratio of 9:9:2, respectively, and the decoloring solution comprised water, methanol, and acetic acid in a volumetric ratio of 7:2:1, respectively (16).

#### 2.7.2 The determination of free amino acids

FAA extraction was conducted as previously described with some modifications (17). The minced pork (2 g) was homogenized with 15% TCA solution (15 ml) at 7000 RPM for 2 min, followed by vibration extraction for 2 h. Subsequently, the homogenate extract was centrifuged at 10,000 RPM for 15 min, and the pH of the supernatant was adjusted to 2–3 using the NaOH solution (5 M). Finally, the 5 ml supernatant was diluted with deionized water to 10 mL and filtered using a 0.22-μm membrane filter. The filtrates were used for FAA detection.

An amino acid analyzer (HT-1010, Tsingtao HiTech Innovative Science and Technology Co., Ltd, Shandong, China) was used to determine the types and contents of FAAs. Each FAA was separated on a Na system HR cation exchange column (125 × 4.6 mm, Tsingtao HiTech Innovative Science and Technology Co., Ltd). The parameters of the amino acid analyzer were as follows: derivative reagent, ninhydrin; reactor temperature, 115°C, column temperature, 50°C; reactor pressure, 10 bar; column pressure, 30 bar; and injection volume, 20 μl. The gradient elution was set as follows: sodium reagent A for 0–22 min; sodium reagent B for 22–33 min; sodium reagent C for 33–51.5 min; sodium reagent D for 51.5–128 min; 0.8% NaOH for 128–136 min; and sodium reagent A for 136–140 min (the components of each mobile phase are shown in Supplementary Table S2).

The internal standard was a sample consisting of 18 types of hydrolyzed amino acids (100 nM). The results were quantified using the internal standard method. The concentration range of the sample solution was 0–2000 nM, and if necessary, samples were diluted to fit this range.

#### 2.7.3 The determination of BAs

The minced pork (5 g) was homogenized with 5% TCA solution (10 ml), and the BAs were extracted via vibration for 60 min. Subsequently, the mixture was centrifuged at 3,600 RPM for 10 min, and the supernatant was collected as the BA extract. The precipitate was further extracted with 5% TCA solution (10 mL) similar to that for the first step. The supernatant obtained from the double extraction was mixed and diluted with the TCA solution to 25 ml.

Next, the extraction and standard solutions of BAs were derivatized for the pre-column reaction as described by Dabadé et al. (18). First, the samples were filtered using a membrane filter (0.45 μm), and the filtrate was mixed with 2 M NaOH solution (50 μl), NaHCO_3_ saturated solution (1.5 ml), and dansyl chloride (1.0 ml) at 40°C for 45 min in the dark. The mixture was shaken twice during the reaction. Then, 25% aqueous ammonia (0.1 ml) was added into the reaction system and placed in the dark for 30 min, followed by placing it in a water bath at 60°C to remove acetone. The reaction mixture was cooled to 25°C and diluted with acetonitrile to 5 ml. Finally, the samples were filtered using a membrane filter (0.22 μm) for analysis.

The samples were analyzed using high-performance liquid chromatography (K2025, Shandong Wooking Instrument Co., Ltd, Shandong, China). The analytes were separated into C18 columns (15 mm × 4.6 mm × 5 μm). The UV detection wavelength was 254 nm, and the injection volume was 10 μl. The column temperature was set at 40°C. The mobile phase A was chromatographic-grade acetonitrile, and the mobile phase B was 0.1 M ammonium acetate. The BA content of each sample was calculated using the standard curve of BAs (Supplementary Table S3). The concentration range of the sample solution was 0–4000 mg/kg.

### 2.8 Molecular docking

AutoDockTools 1.5.6 was used to investigate the probable optimal configurations between the matrix of the liposome and the emulsion delivery system and CEO. As the main component of CEO, cinnamaldehyde was used as the representative molecule to determine the interaction behavior. The molecular structures of cinnamaldehyde and pectin were obtained from the PubChem database, and the structure of the phospholipid bilayer was obtained from the related website of the membrane force field (http://www.fos.su.se/~sasha/SLipids/Downloads.html). The best binding site was then carried out according to the lowest binding energy and visually analyzed via PyMol.

### 2.9 Antibacterial activity

#### 2.9.1 Minimum inhibitory concentration

As the major spoilage bacteria of meat products, *E. coli* and *S. aureus* were used to determine the antibacterial activity of CEO and its delivery system. The MIC values of CEO, CEO-liposome, and CEO-emulsion were examined as previously described (4). Then, 100 μl (10^5^-10^6^ CFU/ml) of the bacterial suspension was evenly incubated onto the plates in the presence of CEO, CEO-liposome, and CEO-emulsion at varying CEO equivalent concentrations (0.0625, 0.125, 0.25, 0.5, 0.375, and 0.5 μL/ml) at 37°C for 24 h. The least CEO equivalent concentration of the plates without strain growth was considered for the MIC.

#### 2.9.2 Conductivity

A conductivity assay was performed as previously described (19). The bacteria were grown for 24 h in nutrient agar. The culture bacteria were diluted (OD600 = 1.0) with phosphate-buffered saline (PBS) and incubated in PBS containing the MIC for 6 h. Afterward, the supernatant was separated by centrifugation at 4,000 RPM for 5 min and measured using an electrical conductivity meter (DDSJ-308A, Shanghai Yidian Scientific Instruments Co., Ltd).

#### 2.9.3 DNA leakage assay

The bacteria were cultured, and the supernatant was collected similarly to that of the conductivity assay. The OD_260_ value of the supernatant was measured using a microplate reader (Supermax 3100, Shanghai, China).

### 2.10 Statistical analysis

Each assay was conducted in triplicate, and the results expressed as means ± standard error were analyzed using SPSS 21 (IBM, Armonk, USA) and Origin 2021 (Massachusetts, USA). The differences among the mean values were examined using a one-way analysis of variance with a Waller-Duncan's multiple range test (the *p* < 0.05).

## 3 Results and discussion

### 3.1 Chemical analysis

Supplementary Figure S1 shows the sensory evaluation values of cooked minced pork treated with different concentrations of CEO. At a treatment CEO concentration of 0.10%, the reduction of taste and smell values became more rapid. Moreover, the differences in the sensory score in the 0.05% CEO-treatment group and control group were not significant. It showed that 0.05% was the maximum concentration of CEO, which did not have a negative effect on the sensory values of cooked minced pork. Therefore, in subsequent experiments, the minced pork was treated with 0.05% CEOs.

**Figure 1:**
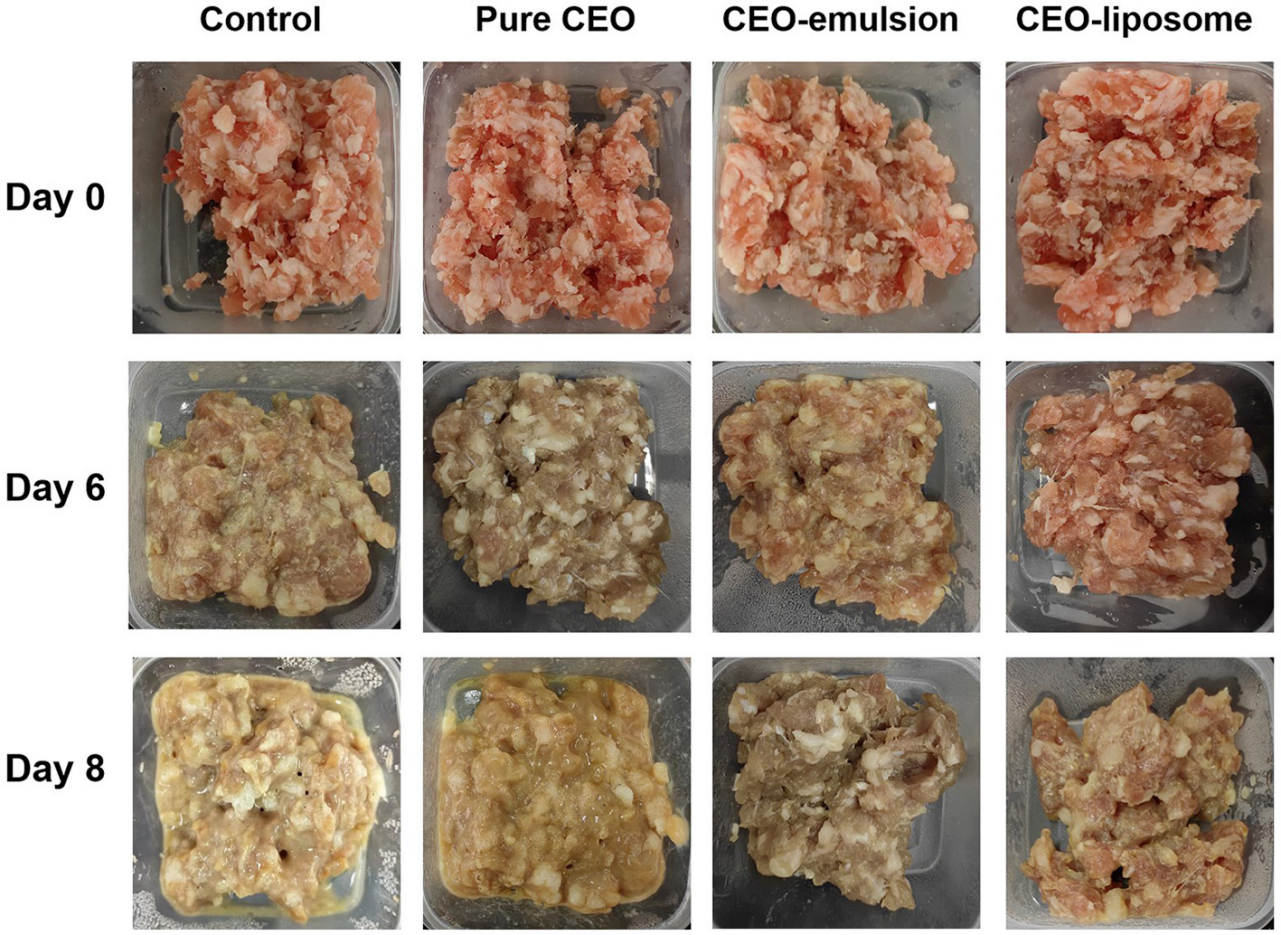
The schematic illustration of minced pork preservation with increasing storage time.

**Figure 2:**
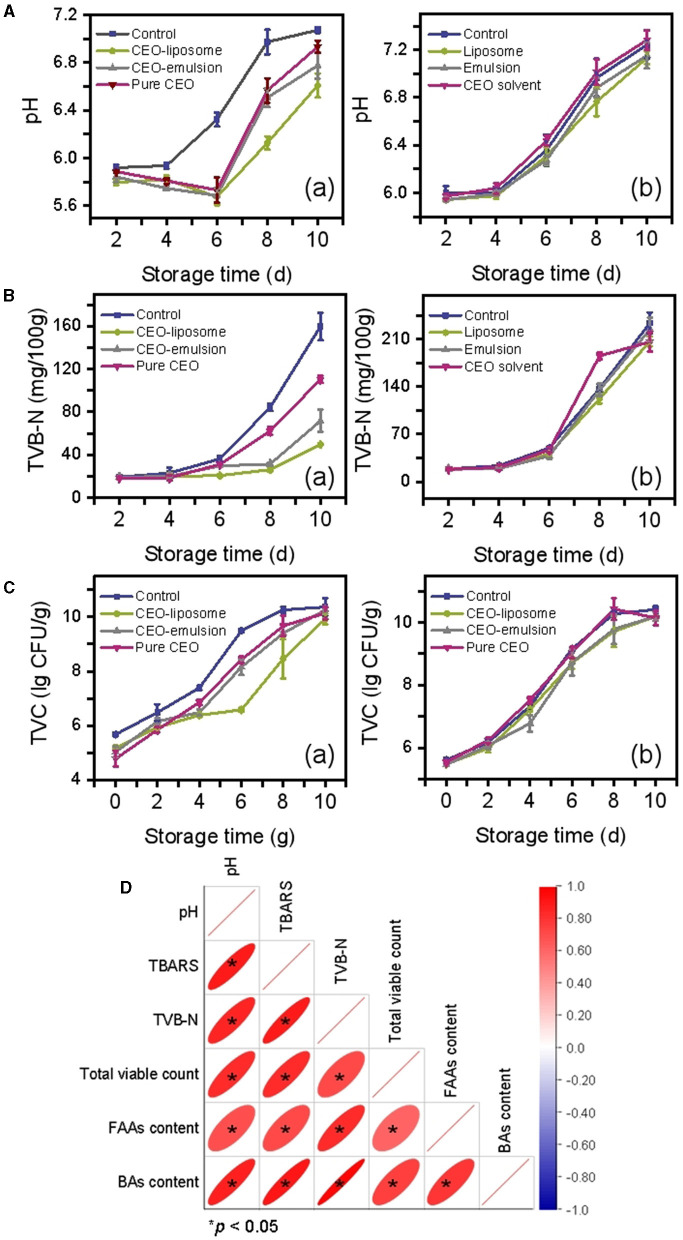
Changes in **(A)** pH, **(B)** TVB-N, **(C)** TVC value of refrigerated mince pork treated with (a) CEOs or (b) without CEOs, and **(D)** Pearson's correlation among physicochemical characteristics.

The schematic illustration of changes in minced pork is shown in Figure 1. The changes in the pH of refrigerated minced pork are shown in Figure 2A (a). The pH of the CEO-emulsion group is 5.87, showing the lowest pH in the initial stage due to the addition of acetic acid used to dissolve chitosan (20). The pH in the control group increased with increased storage time, which was significantly higher than that of the CEO group (*p* < 0.05). Conversely, pH in CEO-liposome, CEO-emulsion, and CEO groups decreased, reaching the lowest value at the storage time of 6 days. The initial decrease in pH can be related to glycogenolysis, decomposition of ATP, and creatine phosphate of muscle tissue (15). The following increase in pH can be attributed to the growth of spoilage microorganisms, which can degrade proteins and produce alkaline substances (21). It is interesting to note that CEO-liposome exhibited the lowest pH value at the storage time of 8 days (pH = 6.61, *p* < 0.05) and the lowest increasing rate among all experimental groups.

To exclude the interference of the delivery system matrix on pH, delivery systems without CEO were also subjected to the same measurement. There was little difference among all matrix groups [Figure 2A (b)]. The emulsion matrix exhibited a slight antibacterial effect on day 4 and day 8, resulting in a slight decrease in pH, but there was no significant difference among all groups after 10-day storage (*p* < 0.05).

TVB-N is an important parameter to evaluate the freshness of meat, which is derived from alkaline nitrogenous substances produced by protein degradation. The freshness decreases with an increase in TVB-N values. The TVB-N values of minced pork increase slowly with the storage time from 0 to 6 days, while they increase rapidly in the storage time of 6–10-day [Figure 2B (a)]. The rapid increase in TVB-N values can be attributed to the growth and proliferation of microorganisms. CEO-liposome exhibited the best effect, followed by CEO-emulsion and pure CEO, which inhibited the formation of amines at the later stage of storage. TVB-N values showed no significant difference among all groups after 10-day storage (*p* < 0.05). In addition, there is no significant difference between the control group and the emulsion matrix of CEO delivery system groups [Figure 2B (b)].

Microorganisms are the major cause of spoilage for most meat products; therefore, TVC values can evaluate the meat spoilage degree. The TVC of minced pork with different treatments was shown in Figure 2C (a), showing that the TVC increased with increased storage time. The initial microorganism count of minced pork treated with CEOs was lower than that of the control, which can be attributed to the antibacterial activity of the CEO. The CEO-emulsion group exhibited the lowest TVC value in the initial stage, probably due to the existence of acetic acid and chitosan.

On the contrary, the CEO-liposome group presented the lowest TVC value from days 6 to 8, showing the strongest antibacterial effect in the tested food model. Additionally, the CEO-emulsion group was greater than that of the pure CEO group from day 4 to day 8. There was no significant difference among all groups on day 10 because the total viable count was saturated.

The TVC of each delivery system matrix is shown in Figure 2C (b). The liposome and emulsion exhibited a slight antibacterial effect due to their distribution, and the emulsion was slightly stronger than the liposome on day 4, probably due to the antibacterial substance in the matrix. However, the antibacterial activity of the emulsion did not last long because the acetic acid was volatile. The differences among all delivery systems without CEOs were small.

The initial pH and TVB-N values were higher than those in the previous report (22). The surface of the pork attracted a great number of microorganisms after slaughter, and the minced state is beneficial for the growth of microorganisms, therefore accelerating protein degradation and the production of alkaline substances. Minced pork treated with CEOs can effectively improve the preservative effect by protecting the volatile components of CEO and control release. The minced pork in the CEO-liposome group extended the longest storage life, followed by the CEO-emulsion and pure CEO groups. The results could be explained as follows.

TVC positively correlated with pH and TVB-N (Figure 2D). CEO cannot maintain antibacterial activity due to its volatile characteristics, causing a decrease in antibacterial effects and alkaline substance production for long-term storage. In addition, CEO can be solubilized in the food lipid matrix, thereby reducing the inhibitory nature of its antibacterial activity against microorganisms in the aqueous phase (23). The state of the liposome aids in its uniform distribution onto the surface of minced pork, increasing the contact area between CEO and microorganisms (24). This reason was also applied to CEO-emulsion, together with the antibacterial activity of acetic acid and chitosan in the emulsion matrix, causing the antibacterial effect of the CEO-emulsion group to be greater than that of the pure CEO group from day 4 to day 8. In addition, liposomes exhibited the best antibacterial effects against the microorganisms in the water phase due to the solvent system. Adding to the phospholipid bilayer, which is similar to the bacterial cell membrane (25), the liposome can comprehensively inhibit the growth of microorganisms in minced pork.

### 3.2 Proteolytic changes analysis

#### 3.2.1 SDS-PAGE

**Figure 3:**
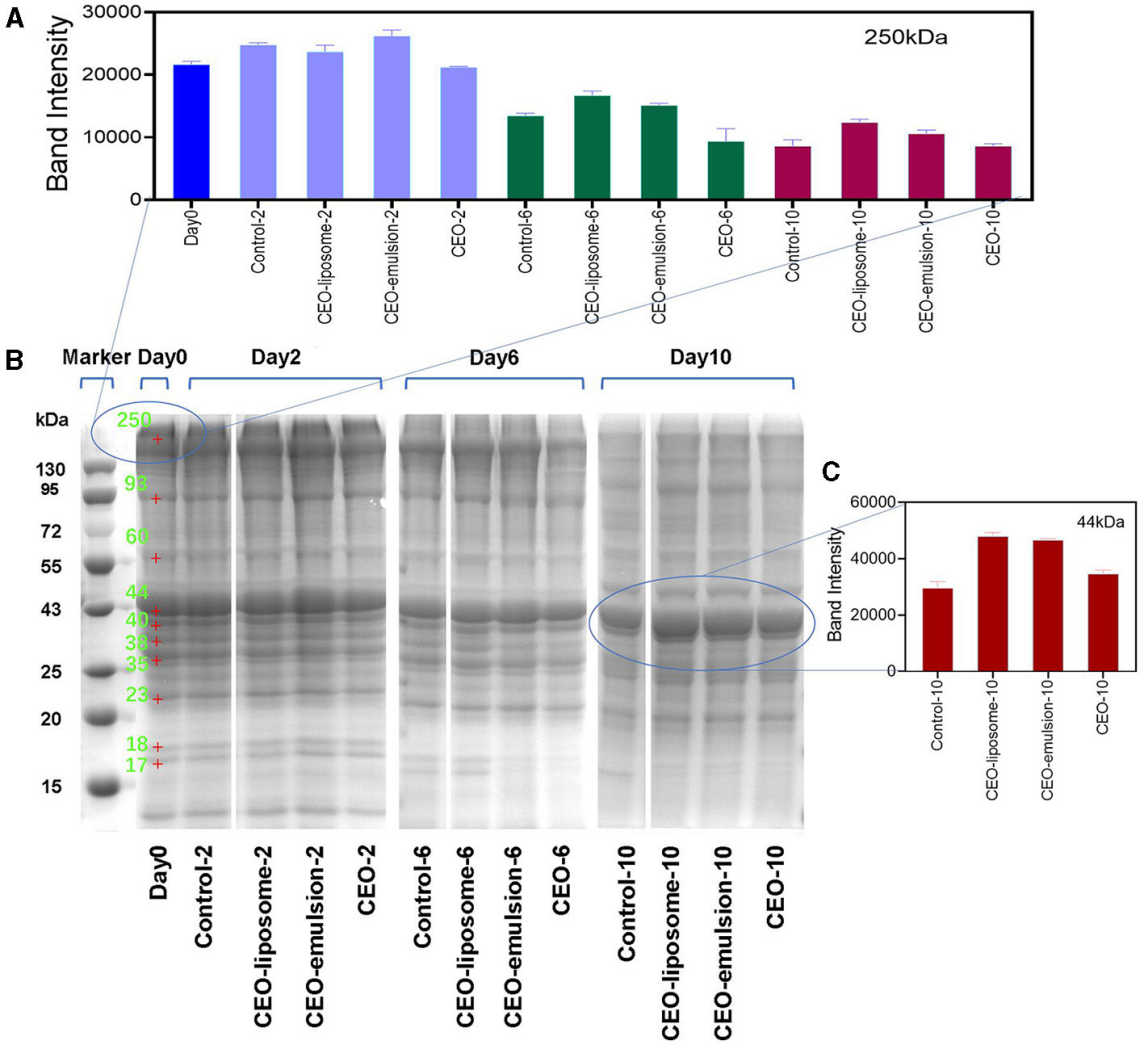
The intensity of the protein bands at **(A)** 250 kDa and **(C)** 44 kDa, and **(B)** 12% SDS-polyacrylamide gel electrophoretic patterns of proteins for refrigerated minced pork.

Protein degradation is an important element of the decline in meat quality. Proteins can be degraded by endogenous enzymes and microorganisms to produce low molecular weight proteins, polypeptides, amino acids, and TVB-N during preservation (26). SDS-PAGE electropherogram showed the changes in minced pork protein content treated with CEOs, as shown in Figure 3. The intensity of nebulin or titin bands (around 250 kDa) and the clear bands with 200–220 kDa in the CEO group significantly decreased on day 6 (Figure 3B), whereas there was no decrease in other groups (27). Combined with the great antibacterial effect of CEO groups (Figure 2C), the decrease in intensity of protein bands can be attributed to the direct action of the exposed CEO on the protein, which can be regarded as degradation (28). In contrast, the delivery system of the CEO-liposome and CEO-emulsion reduced the impacts of CEO to proteins in minced pork.

With increased storage time, the intensity of protein bands decreased due to the proteins hydrolyzed by endogenous enzymes and the reproduction of microorganisms. The intensity of protein bands at 250 kDa decreased, probably due to the degradation of nebulin (Figures 3A, B). The intensity of bands in the region of 108–120 kDa increased (Supplementary Figure S2), which may be generated by myosin degradation. Moreover, the intensity of bands around 35 kDa decreased first, and then the new bands appeared (Figure 3B). The decrease could be attributed to the degradation of myofibrillar protein fractions (27), and the new bands might be derived from the degradation of proteins with high molecular weight, thereby producing low molecular weight proteins or polypeptides.

Abundant proteins were degraded during the 10-day storage. High molecular weight proteins of the control and CEO groups on day 0 almost completely disappeared after 10-day storage, and the low molecular weight protein bands also migrated. The migrated bands might not be derived from the original protein, whereas it was attributed to the degradation of high molecular weight proteins. In particular, the intensity of bands of ~25–29 kDa in the control group on day 10 was higher than that in the other groups. The result can be attributed to the hydrolysis of myofibrillar proteins, causing an increase in the concentration of polypeptides. Furthermore, the low molecular weight proteins or polypeptide bands at 17–18 kDa involving troponins and myosin light chains in all groups gradually disappeared on day 6, which may be due to the FAAs produced by abundant microorganisms. CEO-liposome displayed the strongest intensity of protein bands at 250 kDa on days 6 and 10, proving that it can effectively inhibit the degradation of low molecular weight proteins.

#### 3.2.2 Correlation between biogenic amine and free amino acid

**Figure 4:**
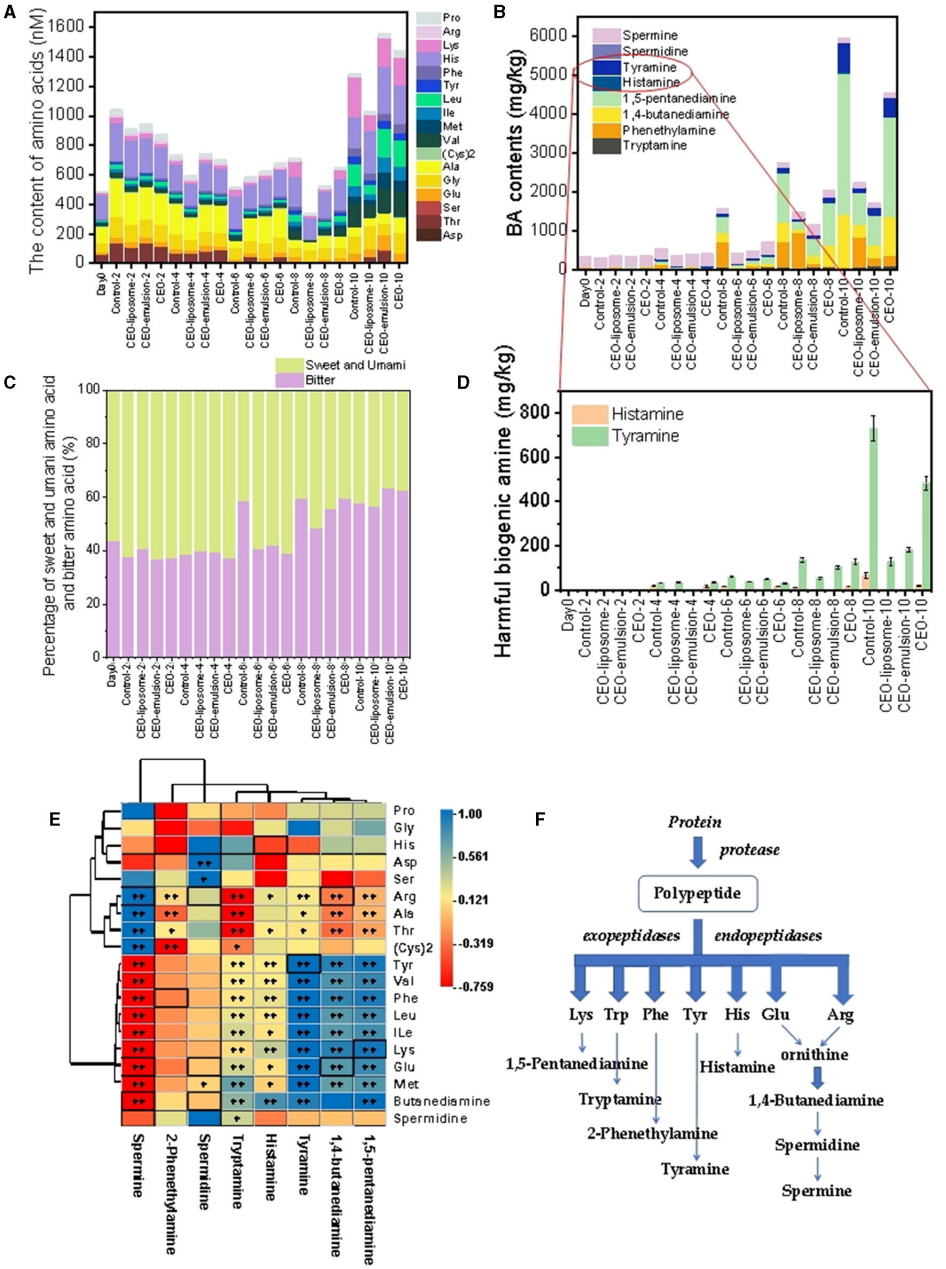
Changes in **(A)** amino acids; **(B)** biogenic amines; **(C)** percentage of sweet and umami amino acids and bitter amino acids; **(D)** harmful biogenic amines of minced pork during storage in refrigeration; **(E)** correlation analysis between amino acids and biogenic amines in minced pork; and **(F)** biogenic amine production process.

FAAs are one of the most important sources contributing to meat flavor. The FAA content in meat will gradually increase after slaughter, especially in meat contaminated by severe microorganism infestations and rotten meat. Therefore, the determination of FAA content is beneficial for evaluating the quality of minced pork. In this assay, 17 amino acids were detected in the CEO-emulsion and pure CEO groups, whereas aspartate was not detected in the control and CEO-liposome groups (Figure 4A). The FAAs on day 0 mainly comprised histidine, alanine, and threonine. Subsequently, the FAA contents increased first and then slightly decreased and finally significantly increased. The FAA contents of all tested groups on day 8 decreased in the following order: control > CEO > CEO-emulsion > CEO-liposome. In addition, there was no significant difference in FAA content in all groups on day 10, proving that proteins were almost completely hydrolyzed.

CEO-liposome maintained the lowest FAA content during the late storage period, which might decrease the proteolysis rate via microorganism inhibition. In addition, Subsection 3.1 indicated that treatment with CEO-liposome can effectively alleviate the changes in pH. Silva et al. (29) highlighted that low pH can increase the amino acid decarboxylase activities. Thus, FAAs derived from proteolysis can transfer to BAs more rapidly, which may be one of the reasons for low FAA content in the CEO-liposome group.

The initial increase in FAAs was probably due to the hydrolysis of minced pork protein by endogenous enzymes, followed by hydrolysis with peptidase and a few microorganisms, thereby producing FAAs as the main components of flavor. For instance, the relative content of umami-taste and sweet-taste characteristic amino acids remains at a high level from day 0 to day 4 (30) (Figure 4C). The following decrease could be explained due to the protein decomposition rate that might be lower than the BA transformation rate due to the increase in microorganisms, which can transfer FAAs to corresponding BAs. As the microorganisms rapidly grow and multiply, the proteolysis was accelerated, causing an increase in some bitter amino acids such as valine, isoleucine, leucine, and lysine, which were present in low quantities in the initial storage. More importantly, as the ratio of bitter amino acids increased, especially in the control group, it resulted in an unpleasant flavor in minced pork (Figure 4C). CEO-liposome exhibited lower bitter amino acid levels than the CEO-emulsion group on day 10, proving that CEO-liposome can better protect the pleasant flavor of minced pork. The ratio of bitter amino acids has no significant difference on day 10 among all groups. Meanwhile, the accelerated proteolysis increased the FAA content on day 10 probably because the protein decomposition rate was higher than that of the decarboxylation of amino acids at this time (30).

BAs are important aromatic components in foods and the potential precursors of carcinogenic substances called nitrosamines, and BAs are produced by the action of microbial-produced amino acid decarboxylation on amino acids. Excessive BA content may convert into toxic metabolites, and most cases of food poisoning caused by BAs can be attributed to the presence of histamine and tyramine (18). Thus, the statistics and analysis of BAs may be an effective index to evaluate the effect of alleviating minced pork spoilage and proteolytic changes under treatment with CEOs. A total of eight BAs were detected in all experiment groups, and the initial storage stage of minced pork comprised spermine and 2-phenethylamine (Figure 4B). With the increased storage time, BA contents of all groups increased, which can be attributed to the reproduction of microorganisms. The whole content of BAs decreased on day 10 of storage as follows: control > CEO > CEO-liposome ≈ CEO-emulsion.

The precursor of BAs was FAAs, which played an important role in the production of BAs. A statistical correlation analysis between FAAs and BAs of minced pork treated with CEOs can provide significant guidance on the modulation of BA production (Figures 4E, F). The BA production process is shown in Figure 4D. The correlation of BAs and their corresponding precursor was as follows: spermine exhibited a significantly positive correction with arginine and a negative correlation with spermidine and glutamate (*p* < 0.01); tyramine was positively associated with tyrosine (*p* < 0.01); 1,4-butanediamine was positively correlated with glutamate and negatively correlated with arginine (*p* < 0.01); 1,5-pentanediamine had a significantly positive correlation with lysine (*p* < 0.01). The result was consistent with that of Fan et al. (31), indicating that the types and contents of BAs were positively correlated with those of FAAs. However, there was no significant difference between some BAs and FAAs, for example, histamine and histidine (Figure 4C). This finding can be attributed to the different inhibitory effects of CEOs on individual amino acid decarboxylase through the release mode and distribution.

Compared to other groups, the BA production in the CEO group tended to increase during day 6 and day 8 markedly due to its chemical instability, indicating CEO showed a greater effect in inhibiting the production of BAs before this time. In addition, CEO-liposome and CEO-emulsion can effectively alleviate the production of FAAs and the formation of BAs because their antibacterial activity is greater than that of pure CEO. Especially, histamine, which has a great amount of histidine as the precursor, was not detected in the two groups. CEO-liposome exhibited a better inhibitory effect on the production of tyramine than CEO-emulsion. Moreover, the distribution and morphology of CEO-liposome and CEO-emulsion increased their contact with amino acid decarboxylase, which can be inhibited by essential oil, resulting in the low increasing extension and total amount of BAs. As a result, CEO-liposome and CEO-emulsion can effectively inhibit the production of histamine and tyramine. Furthermore, the particle size of CEO-liposome and CEO-emulsion was 101.4 nm and 189.6 nm, respectively, which were similar to those in previous studies (13, 14). The smaller the particle size, the more significant the interaction between nanoparticles and the surrounding environment. The larger surface of the CEO-liposome has greater surface energy, which can reduce the aggregation of nanoparticles, improve their stability, and facilitate interface diffusion. Therefore, the interface diffusion of molecules in the CEO-liposome was faster in minced pork due to the smaller nanoscale and more effective in acting on amino acid decarboxylases.

### 3.3 Molecular docking of CEO-liposome and CEO-emulsion

**Figure 5:**
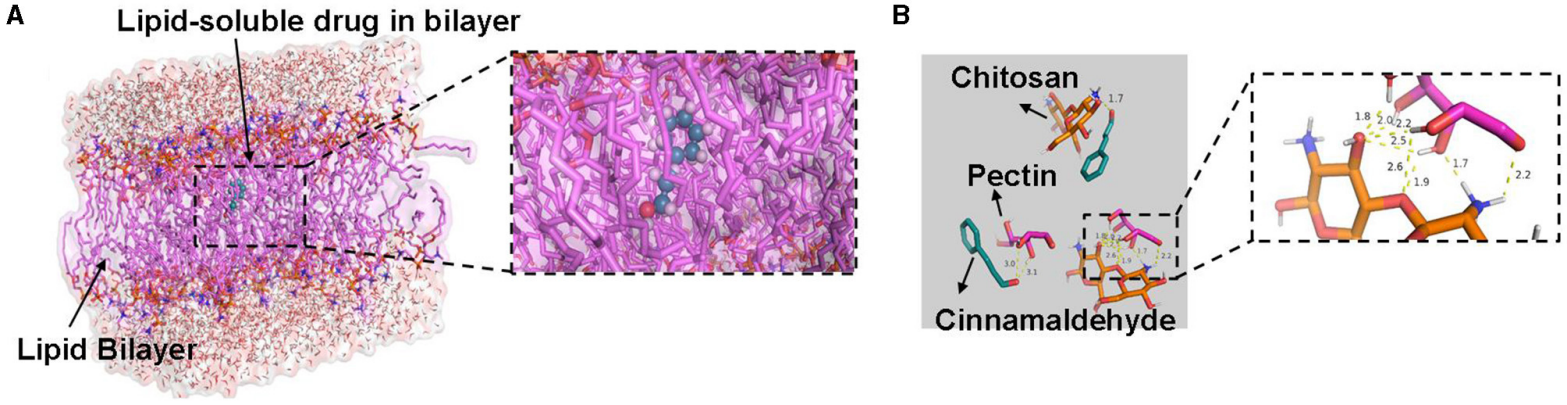
Docking images of cinnamaldehyde over **(A)** phospholipids and **(B)** chitosan and pectin.

Figure 5 illustrates the best binding conformations and possible hydrogen bonding between the main substances of each delivery system and cinnamaldehyde. Phospholipids can spontaneously form a phospholipid bilayer in water, in which liposoluble components are encapsulated. The result showed that cinnamaldehyde did not form hydrogen bonds with the phospholipid bilayer, whereas the phospholipid bilayer encapsulated the cinnamaldehyde molecule through their hydrophobicity (Figure 5A). Pectin and chitosan formed a stable water phase system via multi-hydrogen bond formation in the CEO emulsion. Although the water phase and oil phase formed a stable dispersion system by using the surfactant, the components of CEO formed possible hydrogen bonds with pectin and chitosan, respectively, which further stabilized the loading system (Figure 5B). In brief, the emulsion delivery system can stably load all components of CEO, and the space of the phospholipid bilayer in the liposome was enough for all the hydrophobic components of CEO, which were the major components to exert biological activity.

### 3.4 Antibacterial activity against major spoilage bacteria of meat products

**Figure 6:**
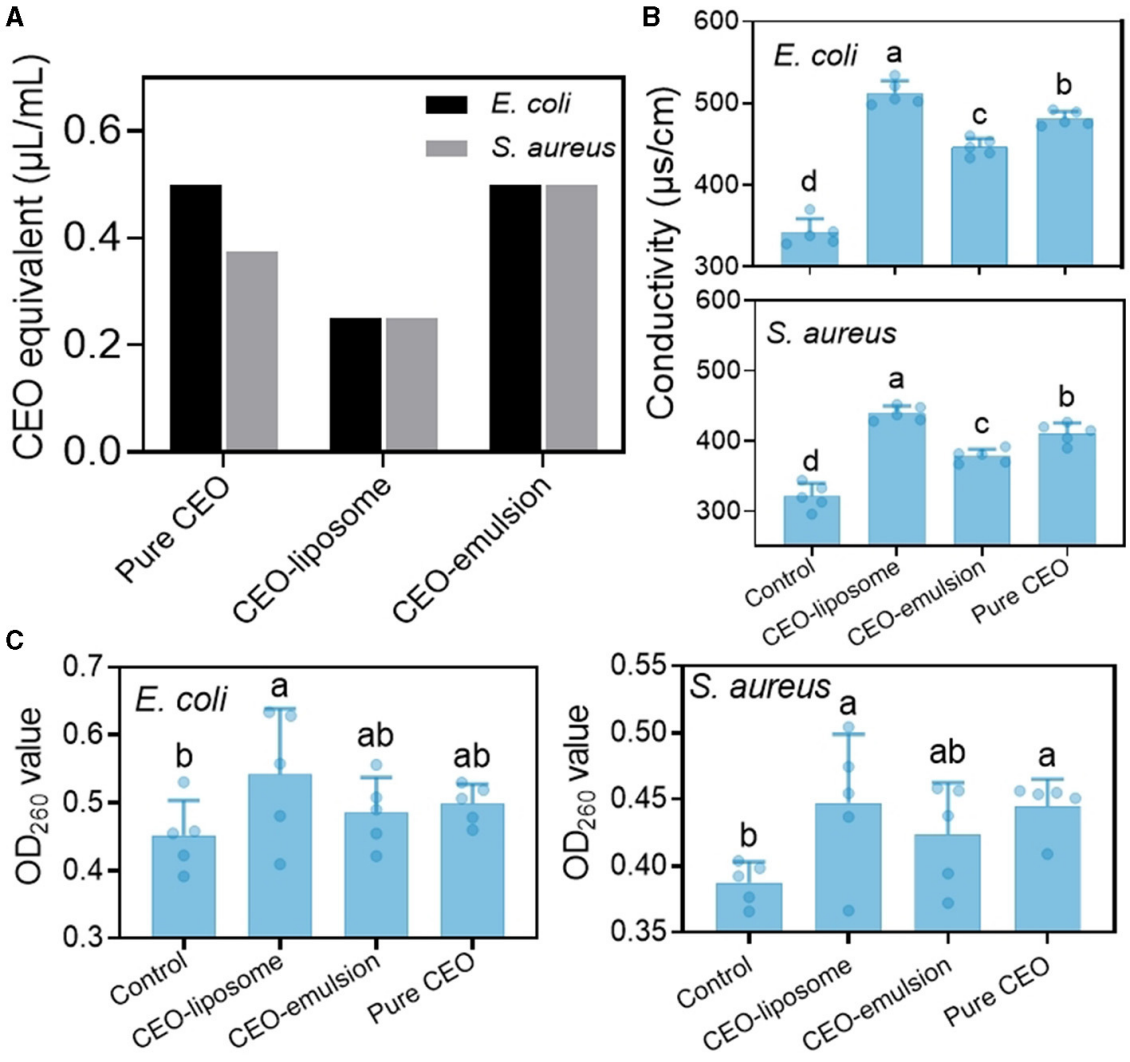
**(A)** MIC value of CEO, CEO-liposome, and CEO-emulsion and the effect of CEO and its delivery system on the **(B)** conductivity and **(C)** OD260 value. Different letters (a–d) indicate statistically significant (*p* < 0.05) differences among samples.

CEO, CEO-liposome, and CEO-emulsion exhibited an inhibitory effect on *E. coli* and *S. aureus*, and CEO-liposome showed stronger antibacterial activity against *S. aureus* than *E. coli*. CEO showed the strongest antibacterial effect among all groups (Figure 6A and Supplementary Figure S3). Terpenes, alcohols, acids, and aldehyde are the main antibacterial compounds of essential oils. As shown in Supplementary Table S5, the main components of CEO are cinnamaldehyde (82.31%) and 2-methoxycinnamaldehyde (9.20%), which inhibit the growth of food pathogenic bacteria through multiple antibacterial targets.

The permeability of the cell membrane is important to cell function. The destruction of the cell membrane can increase the permeability of the cell membrane, resulting in the leakage of intracellular ions and an increase in conductivity (32). Figure 6B shows the conductivity of *E. coli* and *S. aureus* treated with CEO and CEO delivery systems. Both strains had similar changes after treatment, and the conductivity decreased in the following order: CEO-liposome > pure CEO > CEO-emulsion > control.

Nucleic acid cannot travel across the cell membrane due to its high molecular weight, and there is no nucleic acid carrier on the cell membrane. Therefore, the leakage of nucleic acids indicates the changes in cell membrane integrity (32). The OD_260_ value of the two strains was the lowest for the control, and the highest OD_260_ value was attained in the CEO-liposome group. CEO and CEO-emulsion groups showed no significant difference against *E. coli* (Figure 6C). However, the OD_260_ value in the CEO group was greater than that in the CEO-emulsion group against *S. aureus*.

CEO can decrease membrane fluidity and increase membrane hardness. Moreover, the accumulation of CEO can cause cell membrane acidification and protein denaturation, resulting in irreversible membrane injury (33). Liposomes have great compatibility with the bacterial cell membrane; therefore, the structure of the CEO-liposome can aid the CEO to act on bacterial cells. The CEO-liposome displayed the strongest destructive ability on membrane permeability, thereby promoting the leakage of intracellular ions and nucleic acid. Otherwise, the antibacterial capabilities against *E. coli* of CEO and CEO-emulsion were fair. CEO showed a greater antibacterial effect against *S. aureus* than CEO emulsion. This finding was because *S. aureus* is a Gram-positive bacteria, which has a cell wall mainly composed of peptidoglycan. Peptidoglycan is a web-like structure, which allows easy penetration of essential oils and destroys cellular function. The interaction of the substances in CEO-emulsion can aid CEO to become more stable, but it dissolves CEO in the solution system, therefore reducing the reaction to the outside and the antibacterial effect on *S. aureus*.

## 4 Conclusion

This study compared the liposome and emulsion system with CEO based on the quality and proteolysis of refrigerated minced pork. The results indicated that the liposome and emulsion delivery system exhibited strong protection of CEO, extending their properties of preservative effect in minced pork. CEO-liposome was more effective than CEO-emulsion in decreasing microorganism-induced quality deterioration during the 10-day storage, presenting low pH, TVB-N, and TVC values. CEO-liposome had a stronger inhibitory effect against major spoilage bacteria of meat products than CEO-emulsion. This finding was achieved through an increase in cell membrane permeability, leading to an increase in conductivity and the leakage of nucleic acid. It enabled the controlled release of bioactive compounds distributed uniformly on the surface of minced pork and better diffusion. Moreover, we gained a more comprehensive view of the correlation between amino acids and biogenic amine production under treatment with the liposome and emulsion system loading CEO. CEO-liposome was more effective in alleviating the unpleasant flavor and reducing the production of hazardous compounds than CEO-emulsion.

## Data availability statement

The original contributions presented in the study are included in the article/Supplementary material, further inquiries can be directed to the corresponding author.

## Author contributions

KW: Conceptualization, Supervision, Writing – original draft, Writing – review & editing. TZ: Data curation, Investigation, Methodology, Software, Writing – original draft, Writing – review & editing. XC: Project administration, Visualization, Writing – review & editing. PW: Data curation, Formal analysis, Software, Visualization, Writing – review & editing. XD: Software, Supervision, Writing – review & editing.
