# Supplementary material for: Comparative study of a liposome and emulsion system with cinnamon essential oil on the quality and proteolysis of refrigerated minced pork
Source: Front Nutr. 2024 May 3;11:1341827. doi: 10.3389/fnut.2024.1341827 (PMC11100331; doi:10.3389/fnut.2024.1341827)
Supplement: Supplementary file 1 [file Data_Sheet_1.docx]

Supplementary Material

Comparative study of liposome and emulsion system with cinnamon essential oil on the quality and proteolysis of refrigerated minced pork

Kegang Wu^1^, Tong Zhang^1^, Xianghua Chai^1^, Pingping Wang^1^, Xuejuan Duan^1*^

*** Correspondence:** Xuejuan Duan: wandxjbb@163.com

# Supplementary Figures and Tables

## Supplementary Tables

Table S1 Results of CEO-ICs, CEO-liposome, and CEO-emulsion *^a^*.

| Sample | CEO Load (mg/g) |
| --- | --- |
| CEO-liposome | 9.32±0.26^a^ |
| CEO-emulsion | 9.82±1.12^a^ |

*^a^* Values are presented as the mean ± standard deviation. Means in the same column with different superscripts are significantly different (p < 0.05).

Table S2. Mobile phase reagents of amino acid analyzer

|  | Sodium reagent A | Sodium reagent B | Sodium reagent C | Sodium reagent D |
| --- | --- | --- | --- | --- |
| Na^+^ (M) | 0.09 | 0.2 | 0.3 | 0.3 |
| Sodium acetate trihydrate (g) | 12.2 | 27.0 | 41.5 |  |
| Formic acid (mL) | 5.0 | 5.0 | 5.0 |  |
| Acetic acid (mL) | 10.0 | 10.0 | 10.0 |  |
| Ethanol (mL) | 60.0 |  |  |  |
| Trisodium citrate dihydrate (g) |  |  |  | 19.6 |
| Citric Acid Monohydrate (g) |  |  |  | 0.7 |
| Boric acid (g) |  |  |  | 6.0 |
| Sodium hydroxide (g) |  |  |  | 4.0 |
| EDTA. 2Na |  |  |  | 0.5 |
| Constant volume | 1000 | 1000 | 1000 | 1000 |
| pH | 3.33 | 3.30 | 4.00 | 10.45 |

Note: All mobile phase reagents were filtered through 0.45 μm filter.

Table S3. Standard curves of biogenic amine

|  | Standard Curve | Coefficient of determination (R^2^) |
| --- | --- | --- |
| Tryptamine | y=7.64952*x-4.63075 | 0.99986234 |
| Phenethylamine | y=6.84987*x-2.97624 | 0.99998006 |
| 1,4-butanediamine | y=21.28082*x+8.41475 | 0.99995756 |
| 1,5-pentanediamine | y=15.70993*x-4.00738 | 0.9999505 |
| Histamine | y=13.47197*x-1.70446 | 0.99987212 |
| Tyramine | y=13.84158*x-8.13585 | 0.9999484 |
| Spermidine | y=18.85310*x+25.67083 | 0.99771908 |
| Spermine | y=12.33507*x-2.00316 | 0.99912769 |

## Supplementary Methods

S1.2.1 E-nose analysis

E-nose with a PEN3 system was used to evaluate the odor changes of mince pork as previously described (Gao et al., 2017). Briefly, minced pork (2 g) was added into a 40 mL headspace injection bottle and heated in a water bath of 50 °C for 10 min for analysis. The parameters of E-nose were as follows: flush time, 80 s; pre-sampling time, 5s; measurement time, 100 s; and chamber flow, 450 mL/min. The aroma characteristics of each sample was discriminated through the response values of the sensors (**Table S4**).

Table S4 Performance description of sensors for E-nose.

| Array No. | Sensor name | Performance description |
| --- | --- | --- |
| S1 | W1C | Sensitive to aromatic benzene |
| S2 | W5S | Sensitive to nitrogen oxides, especially negative nitrogen oxides |
| S3 | W3C | Ammonia, sensitive to aromatic components |
| S4 | W6S | Mainly selective to hydrides |
| S5 | W5C | Short-chain alkanes, aromatic compounds sensitive |
| S6 | W1S | Sensitive to methyls |
| S7 | W1W | Sensitive to inorganic sulfides and terpenes |
| S8 | W2S | Sensitive to alcohols, aldehydes, and ketones |
| S9 | W2W | Aromatic ingredients, sensitive to organic sulfur compounds |
| S10 | W3S | Sensitive to long-chain alkanes |

## Supplementary Figures

**Supplementary Figure 1.** Sensory evaluation of minced pork treated with different concentrations of CEO. Different letters (a-b) indicate significant difference (*p* < 0.05).

**Supplementary Figure 2.** 8% SDS-Polyacrylamide Gel Electrophoretic patterns of proteins for refrigerated minced pork in day 0, 2, 6, and 10.

Table S5 Volatile components and their relative contents in CEO

Adapted with permission from [MDPI](https://www.mdpi.com/2304-8158/12/1/45), licensed under [CC BY 4.0](https://creativecommons.org/licenses/by/4.0/deed.en)

| **Serial Number** | **Compound Name**  **-Molecular Formula** | **Relative Content (%)** |
| --- | --- | --- |
| 1 | Styrene-C_8_H_8_ | 0.146 |
| 2 | *α*-Pinene-C_10_H_16_ | 0.769 |
| 3 | Camphene-C_10_H_16_ | 0.060 |
| 4 | Benzaldehyde-C_7_H_6_O | 0.864 |
| 5 | *β*-pinene-C_10_H_16_ | 0.033 |
| 6 | p-Cymene-C_10_H_14_ | 0.042 |
| 7 | Limonene-C_10_H_16_ | 0.036 |
| 8 | Salicylal-C_7_H_6_O_2_ | 0.221 |
| 9 | Acetophenone-C_8_H_8_O | 0.039 |
| 10 | Phenylethyl alcohol-C_8_H_10_O | 0.471 |
| 11 | Benzenepropanal-C_9_H_10_O | 0.640 |
| 12 | ((1S)-endo)-(-)-borneol-C_10_H_16_O | 0.133 |
| 13 | *α*-Terpineol-C_10_H_18_O | 0.031 |
| 14 | 2-Methoxybenzaldehyde- C_8_H_8_O_2_ | 0.667 |
| 15 | Acetic acid 2-phenylethyl ester-C_10_H_12_O_2_ | 0.098 |
| 16 | (E)-Cinnamaldehyde-C_9_H_8_O | 82.306 |
| 17 | 3-Phenylprop-2-en-1-ol-C_9_H_10_O | 0.112 |
| 18 | Caryophyllene-C_15_H_24_ | 0.142 |
| 19 | (E)-alpha-bergamotene-C_15_H_24_ | 0.074 |
| 20 | Coumarin-C_9_H_6_O_2_ | 0.845 |
| 21 | (2-Nitroprop-1-en-1-yl)benzene-C_9_H_9_NO_2_ | 0.973 |
| 22 | 2-Methoxycinnamaldehyde-C_10_H_10_O_2_ | 9.199 |
| 23 | Gamma-muurolene-C_15_H_24_ | 0.189 |
| 24 | *α*-curcumene-C_15_H_22_ | 0.158 |
| 25 | *α*-muurolene-C_15_H_24_ | 0.146 |
| 26 | *β*-bisabolene-C_15_H_24_ | 0.159 |
| 27 | (+)-δ-cadinene-C_15_H_24_ | 0.320 |
| 28 | Nerolidol-C_15_H_26_O | 0.189 |
| 29 | Spathulenol-C_15_H_24_O | 0.163 |
| 30 | Caryophyllene oxide-C_15_H_24_O | 0.149 |
| 31 | alpha-Bisabolol-C_15_H_26_O | 0.062 |
| 32 | Myristic acid-C_14_H_28_O_2_ | 0.095 |
| 33 | Benzyl benzoate-C_14_H_12_O_2_ | 0.081 |
| 34 | Pentadecanal-C_15_H_30_O | 0.020 |
| 35 | Pentadecanoic acid -  C_15_H_30_O_2_ | 0.042 |
| 36 | Phenethyl benzoate-C_15_H_14_O_2_ | 0.055 |
| 37 | Palmitic acid-C_16_H_32_O_2_ | 0.241 |
| 38 | Phytol-C_20_H_40_O | 0.017 |
| 39 | Anethole-C_10_H_12_O | 0.015 |


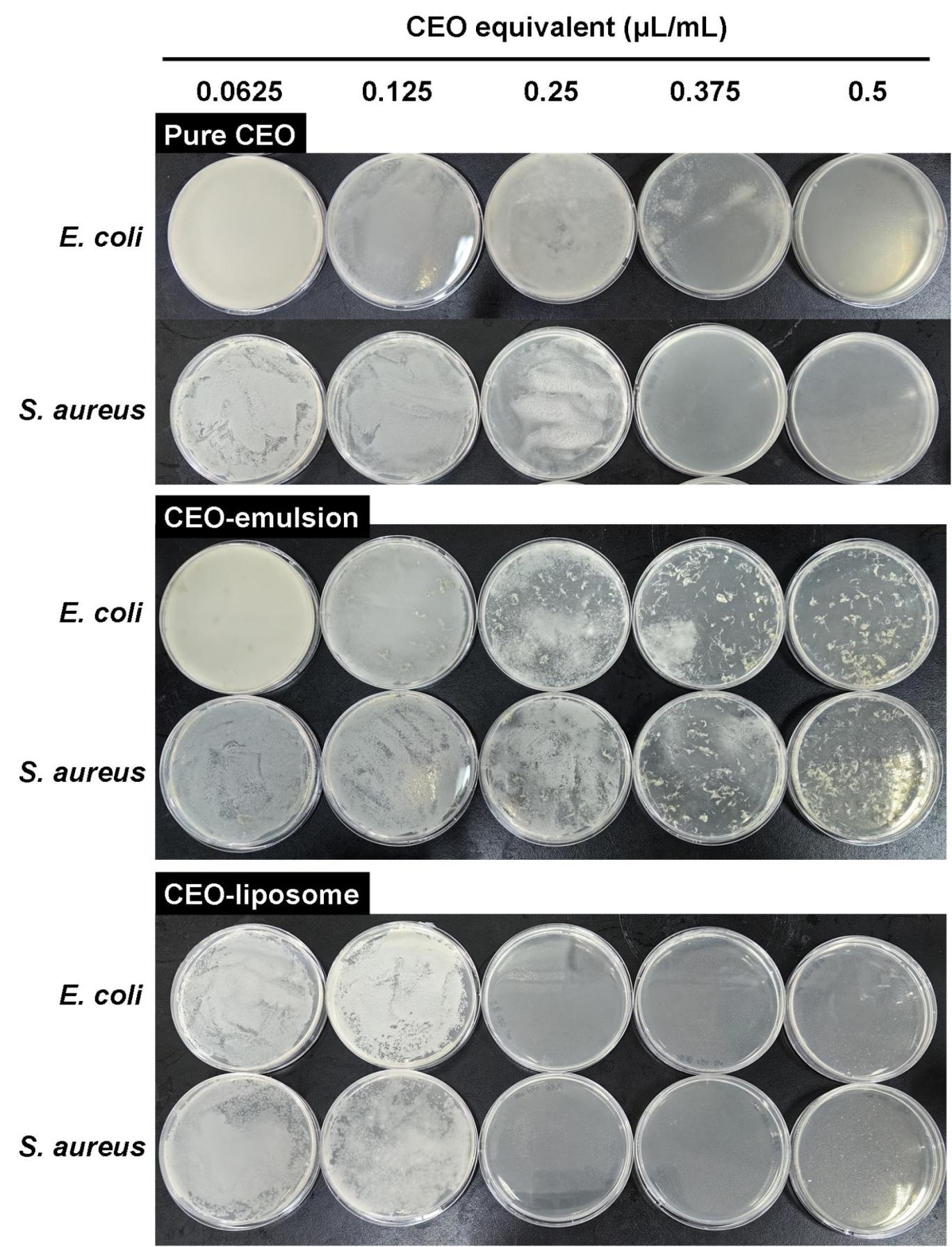


**Supplementary Figure 3.** Minimum inhibitory concentration of CEO, CEO-liposome, and CEO-emulsion.
